# Nurse Practice Environment, Job Satisfaction, and Turnover and Patient Falls

**DOI:** 10.1001/jamanetworkopen.2025.51223

**Published:** 2025-12-29

**Authors:** Kaili Mauricio, Amy L. Bulger, Corrine Y. Jurgens, Brielle Ruscitti, Katherine E. Gregory, Diana Bowser

**Affiliations:** 1 William F. Connell School of Nursing, Boston College, Chestnut Hill, Massachusetts

## Abstract

**Importance:**

Falls in hospitalized patients are common adverse events with substantial consequences for patient safety and hospital costs. Understanding modifiable organizational factors associated with falls is essential for prevention.

**Objective:**

To examine whether nurse job satisfaction, practice environment, and turnover are associated with patient falls.

**Design, Setting, and Participants:**

This cross-sectional study examined US acute care hospital unit-level data from the National Database of Nursing Quality Indicators between January 1, 2022, and December 31, 2023. Unit types included medical, surgical, and medical-surgical combined. Data were received on December 20, 2024, and analyzed from January 6 to April 30, 2025.

**Exposures:**

Nurse job satisfaction and practice environment measured at the unit level.

**Main Outcomes and Measures:**

The primary outcome was the reported rate of patient falls per 1000 patient-days. Structural equation modeling was used to evaluate both direct associations of job satisfaction and practice environment and indirect association of nurse turnover with falls.

**Results:**

The cohort comprised 8584 units (2736 medical, 1384 surgical, and 4464 medical-surgical combined), with 3459 units (40.3%) in hospitals with 199 beds or fewer, 2215 (25.8%) in hospitals with 200 to 299 beds, and 3375 units (39.3%) situated in Magnet-designated hospitals. The total number of patient falls from 2022 to 2023 was 42 929, ranging from 12.3% in surgical units to 52.5% in medical-surgical units. High registered nurse and advance practice registered nurse turnover was significantly associated with increased patient falls (β = 0.01; 95% CI, 0.01-0.02). The job satisfaction item nurses’ intent to stay was significantly associated with lower turnover (β = −3.50; 95% CI, −6.25 to −0.75). No other satisfaction or practice environment items were significantly associated with turnover. Indirect associations of job satisfaction or practice environment with regard to falls through nurse turnover were not consistently significant.

**Conclusions and Relevance:**

This cross-sectional study found that higher nurse turnover was associated with increased patient falls. While limited evidence links specific job satisfaction measures to turnover, these findings reinforce the importance of nurse retention in supporting patient safety. Future research should explore targeted strategies to improve retention and reduce falls.

## Key Points

**Question:**

What is the association of nurse job satisfaction, practice environment, and turnover with patient falls?

**Findings:**

In this cross-sectional study of 8584 hospital units in the National Database of Nursing Quality Indicators, higher nurse turnover was significantly associated with increased patient falls. Nurse job satisfaction and the practice environment were indirectly associated with fall rates through their influence on turnover.

**Meaning:**

These findings suggest that improving nurse retention by enhancing job satisfaction and the practice environment may reduce patient falls and improve hospital safety outcomes.

## Teaser

This cross-sectional study examines whether nurse job satisfaction, practice environment, and turnover are associated with inpatient falls in US acute care hospitals.

## Introduction

In the US, upward of 700 000 hospitalized patient falls occur each year,^1^ many of which are classified as preventable hospital-acquired injuries.^2,3^ The cost of falls is estimated to be more than $50 billion for nonfatal falls and $754 million for fatal falls.^4,5^ Falls in the hospital setting are of major concern as the estimated additional cost of an inpatient fall in the hospital ranges between $2680 and $15 491.^4,6^ While several factors have been shown to increase falls, prior research has suggested that organizational factors, such as nurse practice environment and nurse staffing, are key factors in fall rates.^7,8^

Registered nurses (RNs) represent the largest segment of the health care workforce in the US^6^ and are critical to reducing patient falls. Nurse-driven fall prevention interventions have been shown to substantially reduce fall rates.^9,10^ For example, implementing a fall prevention tool kit has been shown to significantly reduce patient falls in hospital medical units.^10^ However, nurse shortages and turnover are growing issues associated with patient outcomes and have additional economic consequences.^11^ In 2023, the US national staff nurse turnover rate was 18.4%, with each RN turnover costing $56 300, on average, resulting in $3.9 to $5.8 million annual turnover costs for an average health care organization.^12^ It is expected that organizational-level nurse turnover will continue to increase. The 2024 National Council of State Boards of Nursing survey found that more than one-quarter of nurses surveyed plan to leave the profession by 2027.^13,14^ A continued increase in organizational-level turnover rates is concerning, with potential impacts on future nurse shortages and, in turn, patients and health care systems.^15^

Prior research has linked nurse turnover to demographic, job satisfaction, organizational, and burnout factors.^16^ Younger and newly graduated RNs have particularly high turnover rates.^16^ Additionally, the COVID-19 pandemic has substantially impacted health care organizations, with long-term effects on the nursing workforce.^17^ Another key organizational factor shown to be associated with nurse retention and turnover is the nurse practice environment.^18^ Standardized measures of demographic, organizational, and environmental factors provide useful data for examining drivers of turnover.

A component of the National Database of Nursing Quality Indicators (NDNQI) is the NDNQI RN Satisfaction Survey, which assesses the practice environment (Practice Environment Scale of the Nursing Work Index [PES-NWI]) and job satisfaction, 2 key components that have been associated with both nurse turnover^18,19^ and patient falls.^20,21,22^ The PES-NWI, developed by Lake,^23^ comprises 5 subscales to measure the nurse practice environment and is the most commonly used scale to assess the practice environment within the US and internationally.^24^ The 5 subscales include nurse participation in hospital affairs, nursing foundations for quality care, nurse manager ability, leadership and support of nurses, staffing and resource adequacy, and collegial nurse-physician collaborations.^23^ Several studies have shown the association between unit-level PES-NWI scores and nurse turnover, suggesting that nursing units with higher PES-NWI scores have lower rates of RN turnover.^18,19^ Furthermore, within the PES-NWI subscales, units with high staffing and resource adequacy scores experience significantly lower RN turnover.^18^

These findings suggest that improvements in practice environments may enhance nurse retention^25^ and, subsequently, influence patient care. However, there is limited research examining the multidimensional association among PES-NWI scores; unit-level turnover; and patient outcomes, including falls. Prior research has noted that higher nurse turnover is associated with higher rates of patient falls.^26,27^ As well, higher nurse job satisfaction has been associated with reduced patient falls.^28^ However, prior analyses have not controlled for other factors, such as PES-NWI scores, or patient-level factors, such as fall risk,^28^ suggesting the need for further investigation.

While there is some initial research that has examined the association between the nurse practice environment and unit-level turnover and patient outcomes,^20,21^ there is limited literature exploring this association between nursing turnover and patient falls, particularly after the COVID-19 pandemic. Research has also noted that nurse job satisfaction has declined following the pandemic,^29^ yet its association with increasing nurse intent to stay, nurse turnover, and potential impacts on patient falls has not been thoroughly examined. Given this limited evidence, the purpose of our investigation was to examine whether patient falls in hospitals are associated with nurse job satisfaction, practice environment, and nurse turnover.

## Methods

### Data Source

This cross-sectional study used 2 databases for the analysis: (1) The NDNQI clinical data collected by Press Ganey Associates LLC from January 1, 2022, to December 31, 2023,^30^ and (2) the NDNQI RN Satisfaction Survey for the same years.^23^ This study was approved by the Boston College Institutional Review Board, which determined it exempt from informed consent because the data were deidentified and collected routinely during quality-improvement operations and not considered human participants research. This study followed the Strengthening the Reporting of Observational Studies in Epidemiology (STROBE) reporting guideline for cross-sectional studies.^31^

The NDNQI clinical survey consists of unit-level nursing-sensitive indicators that measure patient outcomes, nursing workforce characteristics, and the quality of the nurse practice environment collected on a monthly or quarterly basis.^30^ Indicators collected at the level of the hospital unit include measures of patient falls, pressure injuries, nurse staffing levels, nurse job satisfaction, and unit-level nurse turnover rates. The PES-NWI, a widely used tool that assesses nurses’ perceptions of their practice environment, captures key factors such as staffing adequacy, nurse-physician collaboration, and support for nursing leadership.^23^ Respondents to the PES-NWI are RNs or advance practice RNs (APRNs) (full or part time, regardless of job title) who spend at least 50% of their time in direct patient care and have been employed a minimum of 3 months on the unit. Unit-based per-diem RNs employed by the hospital are eligible; agency or contract RNs are not eligible. We refer to our sample of RNs and APRNs as RNs; however, workforce statistics suggest that there is a higher proportion of RNs working in hospital settings.^32,33^ The information in both datasets provide a unique and comprehensive view of nurse practice environment conditions and nursing workforce factors and how they may relate to patient outcomes, making the data particularly useful for investigating the associations between nurse practice environment, job satisfaction, and turnover and a key patient safety indicator, falls. We used items from 2 PES-NWI domains to maximize response rates: foundations of nursing quality (10 questions) and resource adequacy (4 questions). Composite scores were considered, but subscales were analyzed to retain the largest sample.

### Measures

Data from the NDNQI clinical data survey and the NDNQI RN Satisfaction Survey with PES-NWI datasets were merged. The primary dependent variable was patient falls calculated as the quarterly number of falls per 1000 patient-days. The main independent variable was RN and APRN quarterly turnover within each unit. Turnover was defined as the sum of the number of individual RN and APRN staff who left during a quarter divided by the mean number of full- and part-time employed staff during a quarter period, multiplied by 100.

The analysis also controlled for patient-, unit-, and hospital-level factors. Patient-level factors included the mean age of patients who experienced falls and whether the patient was deemed to be at risk for falls. The unit-level factors included the total number of patient falls, the female-to-male patient fall ratio, the unit type (medical, surgical, and medical-surgical combined), the unit nurses’ perceptions of staffing adequacy and resource availability, and the unit nurses’ evaluations of the foundations of quality care (explained in the next section). The nurse job satisfaction measure was based on 7 questions developed specifically for the NDNQI that assessed the degree to which RN respondents were satisfied with their work.^34,35^ Hospital-level factors included the hospitals’ Magnet hospital status as designated by the American Nurses Credentialing Center^36^ and the hospital size based on number of beds.

The PES-NWI comprises 31 questions across 5 categories. Depending on the model used in the statistical analysis, we either used all questions separately or selected PES-NWI categories. Select PES-NWI categories were used to limit the number of missing variables across some categories. All PES-NWI questions were measured on 4-point Likert scales and standardized so that higher scores reflected more positive perspectives. Nurse job satisfaction was a composite total score for all 7 questions measured on a 6-point Likert scale. A full list of the variables and their definitions are included in eTable 1 in Supplement 1. The NDNQI RN Satisfaction Survey also included 7 items that assessed nurses’ job satisfaction, enthusiasm, and general enjoyment with their jobs; perceptions of job quality compared with other RNs; motivation to attend work; and considerations of job switching. We used the item “RNs on our unit would not consider taking another job” to measure nurse intent to stay.

### Structural Equation Modeling

A structural equation modeling (SEM) approach was used to examine how nurse turnover, job satisfaction, and practice environment influenced the number of falls per 1000 patient-days while controlling for other patient-, unit-, and hospital-level variables. To maximize sample size, analyses focused on 2 PES-NWI domains: foundations for quality of care (10 items) and staffing and resource adequacy (4 items). We used SEM to investigate the complex, multidimensional association of nurse turnover, nurse job satisfaction, the multiple PES-NWI categories with patient falls. The SEM was designed to test whether nurse job satisfaction and various PES-NWI variables were indirectly associated with patient falls through nurse turnover, as illustrated by the conceptual framework in eFigure 1 in Supplement 1, with the hypothesized outcomes explained in eAppendix 1 in Supplement 1. In this study, indirect refers specifically to the SEM pathway in which nurse job satisfaction and practice environment influenced patient falls indirectly through their effect on turnover, rather than a direct association with patient falls. This method used all available data and provided unbiased estimates while accounting for both individual-level and group-level variance.^37,38^ We evaluated model fit using residual-based statistics, including the coefficient of determination, due to the use of clustered SEs.

### Statistical Analysis

We used SEM to investigate the complex, multidimensional association of nurse turnover, job satisfaction, and practice environment with unit-level falls per 1000 patient-days. We used the full information maximum likelihood^37^ estimation method in SEM to ensure a robust analysis with the largest number of units in the sample and minimal bias due to missing data. The effective size was 5461 units built on an aggregate of 49 268 falls over the years 2022 and 2023. More information on missing data is provided in eAppendix 2 in Supplement 1. As falls per 1000 patient-days were aggregated, a unit-quarter observation may reflect multiple falls by the same patient or falls across multiple patients; the analysis did not distinguish between individual-level repeat events. To address this potential correlation across repeated quarterly observations within units, we estimated clustered SEs at the unit level and included quarter fixed effects to adjust for temporal trends.

An additional linear regression analysis was performed (eTable 2 in Supplement 1), with the corresponding description shown in eAppendix 3 in Supplement 1. The data were received on December 20, 2024, and analyzed from January 6 to April 30, 2025, using Stata, version 18.5 (StataCorp LLC). A 2-sided *P* < .05 was considered statistically significant.

## Results

Table 1 shows the characteristics of the hospital units included in this analysis. The total number of falls from 2022 to 2023 was 42 929, ranging from 5285 in surgical units to 22 550 in medical-surgical units. Medical units had a mean (SD) of 2.97 (3.89) falls per 1000 patient-days, and surgical units and medical-surgical units had a mean (SD) of 2.39 (2.29) and 2.79 (1.95) falls per 1000 patient-days, respectively. Quarterly mean patient falls per 1000 patient-days are shown in eFigure 2 in Supplement 1. The percentage of patients who were at risk for falls was 45.0% in medical units, 37.1% in surgical units, and 42.5% of patients in medical-surgical units. The mean (SD) RN and APRN turnover rate for full-time employees was 10.8% (13.6%) in medical units, 11.2% (16.7%) in surgical units, and 10.3% (14.2%) in medical-surgical units. Quarterly mean unit turnover rates are also shown in eFigure 2 in Supplement 1. For patient-level characteristics across unit types, the mean (SD) age of patients who fell was 66.7 (9.5) years. The unit-level data comprised 8584 total units with 2736 medical units, 1384 surgical units, and 4464 medical-surgical units.

**Table 1.** Descriptive Characteristics of Units

Characteristic	Medical	Surgical	Medical-surgical combined	Total
**Unit level**				
No. of patient falls	15 094	5285	22 550	42 929
Falls per 1000 patient-days, mean (SD)	2.97 (3.89)	2.39 (2.29)	2.79 (1.95)	2.78 (2.77)
Patients at risk for falls, mean (SD), %	45.0 (40.0)	37.1 (38.0)	42.5 (41.0)	42.4 (40.0)
Unit RN and APRN turnover, mean (SD), %^a^	10.8 (13.6)	11.2 (16.7)	10.3 (14.2)	10.6 (14.5)
Age of patients who fell, mean (SD), y	66.4 (9.2)	66.2 (10.3)	67.0 (9.5)	66.7 (9.5)
Unit female-to-male patient falls ratio	0.90	0.99	0.98	0.95
**Hospital level**				
No. of units	2736	1384	4464	8584
Bed size, No. (%)				
≤199	829 (30.3)	553 (40.0)	2071 (46.4)	3456 (40.3)
200-299	710 (26.0)	331 (23.9)	1170 (26.2)	2213 (25.8)
300-499	735 (26.9)	280 (20.2)	835 (18.7)	1844 (21.5)
≥500	462 (16.9)	220 (15.9)	388 (8.7)	1071 (12.5)
Magnet hospital designation, No. (%)	1163 (42.5)	565 (40.8)	1647 (36.9)	3391 (39.5)

Abbreviations: APRN, advance practice registered nurse; RN, registered nurse.

^a^ Captures the number of RNs and APRNs who left the unit as a percentage of all full-time employees on the unit.

Hospital-level data included bed size category and Magnet hospital status. For bed size category, which captured the hospital size of each of the units analyzed, 3456 units (40.3%) were within hospitals that had 199 beds or fewer and 2213 (25.8%) were within hospitals that had between 200 and 299 beds. A total of 1163 medical units (42.5%), 565 surgical units (40.8%), and 1647 medical-surgical units (36.9%) were situated within hospitals designated as Magnet hospitals.

Table 2 shows the results of the SEM. Among the NDNQI RN items examined (including both PES-NWI and NDNQI RN Satisfaction Survey), only 1 job satisfaction indicator was significantly associated with turnover (β = −3.50; 95% CI, −6.25 to −0.75; *P* = .01). While no other PES-NWI items from the foundations of nursing quality or resource adequacy domains were associated with significant pathways to turnover, they all contributed to the model. Turnover showed a positive association with patient falls (β = 0.01; 95% CI, 0.01-0.02; *P* < .001). Thus, the PES-NWI contributed to the model primarily through the job satisfaction pathway, showing that a nurse turnover increase of 1 percentage point is associated with an increase of 0.01 patient falls per 1000 patient-days. Put another way, a turnover increase of 10 percentage points is associated with approximately 36 additional patient falls per year in a hospital with 1000 inpatients per day, after accounting for patient risk, staffing levels, and unit characteristics. The female-to-male sex ratio (β = 0.22; 95% CI, 0.15-0.28; *P* < .001) and the proportion of at-risk patients (β = 1.22; 95% CI 0.99-1.45; *P* < .001) showed positive associations with fall rates, suggesting that units with a higher proportion of female patients and patients who were classified as a fall risk may be more likely to experience falls. Unit type also had a significant association, with medical units showing higher fall rates compared with other unit types (β = 0.23; 95% CI, 0.03-0.43; *P* = .03). Hospitals with Magnet status had fewer falls (β = −0.15; 95% CI, −0.24 to −0.05; *P* = .002), and larger hospitals (measured by bed size) were associated with lower fall rates (β = −0.11; 95% CI, −0.17 to −0.05; *P* < .001).

**Table 2.** Structural Equation Modeling Examining the Association of Nurse Turnover With Patient Falls

Variable	β Coefficient (95% CI)	*P* value
**Falls per 1000 patient-days**		
RN and APRN turnover	0.01 (0.01 to 0.02)	<.001
Patient age	−0.01 (−0.01 to 0.00)	.09
Female-to-male patient ratio	0.22 (0.15 to 0.28)	<.001
At-risk share	1.22 (0.99 to 1.45)	<.001
Medical unit	0.23 (0.03 to 0.43)	.03
Magnet hospital designation	−0.15 (−0.24 to −0.05)	.002
Bed size category	−0.11 (−0.17 to −0.05)	<.001
Constant	171.98 (−34.35 to 378.30)	.10
**RN and APRN turnover**		
Nurse job satisfaction		
As RNs, we are fairly well satisfied with our jobs on our unit.	−1.14 (−4.67 to 2.39)	.53
RNs on our unit would not consider taking another job.	−3.50 (−6.25 to −0.75)	.01
I have to force myself to come to work much of the time.	1.49 (−0.69 to 3.68)	.18
RNs on our unit are enthusiastic about our work almost every day.	−0.07 (−3.23 to 3.10)	.97
RNs on our unit like our jobs better than the average RN does.	−2.45 (−6.14 to 1.24)	.19
I feel that each day on my job will never end.	0.66 (−1.26 to 2.59)	.50
We find real enjoyment in our work on our unit.	0.52 (−4.67 to 5.71)	.84
Selected PES-NWI subscales		
Foundations of nurse quality		
Active staff development or continuing education programs for nurses	2.20 (−3.87 to 8.27)	.48
High standards of nursing care expected by the administration	5.07 (−0.89 to 11.03)	.10
A clear philosophy of nursing that pervades the patient care environment	−1.53 (−8.53 to 5.47)	.67
Working with nurses who are clinically competent	−3.85 (−9.18 to 1.48)	.16
An active quality assurance program	1.60 (−4.81 to 8.01)	.63
A preceptor program for newly hired RNs	−3.27 (−8.99 to 2.46)	.26
Nursing care based on a nursing, rather than a medical, model	3.53 (−2.06 to 9.12)	.22
Written, up-to-date nursing care plans for all patients	−1.71 (−7.88 to 4.46)	.59
Patient care assignments that foster continuity of care, ie, the same nurse cares for the patient from one day to the next	4.34 (−0.53 to 9.21)	.08
Use of nursing diagnoses	−1.36 (−6.94 to 4.23)	.63
Sufficient resources for nurses		
Adequate support services to allow time with patients	2.80 (−1.67 to 7.26)	.22
Enough time and opportunity to discuss patient care, problems with other nurses	1.42 (−3.47 to 6.30)	.57
Enough RNs to provide quality patient care	−1.72 (−7.32 to 3.88)	.55
Enough staff to get the work done	−0.60 (−6.71 to 5.52)	.85
Constant	4.26 (−8.55 to 17.08)	.52

Abbreviations: APRN, advance practice registered nurse; PES-NWI, Practice Environment Scale of the Nursing Work Index; RN, registered nurse.

Within the structural first stage of the SEM, the proportion of RNs in the unit who would not consider taking another job (nurse intent to stay) was significantly associated with lower turnover (β = −3.50; 95% CI, −6.25 to −0.75; *P* = .01), indicating the importance of retention-related attitudes among nursing staff (Table 2). The SEM showed a moderate fit to the data. The *R*^2^ value for nurse turnover was 0.10, suggesting that 10% of the variance in this outcome was explained by the estimators in the model. Similarly, the *R*^2^ value for falls per 1000 patient-days was also 0.10, suggesting that the estimators explained 10% of the variance in falls.

## Discussion

This cross-sectional study examined whether nurse turnover, patient characteristics, and unit- and hospital-level characteristics are associated with patient fall rates. We found that higher nurse turnover was associated with increased patient falls, underscoring the role of nurse retention in ensuring patient safety. These findings stress the need to consider organizational characteristics when addressing staff turnover and patient falls.

Our findings align with prior research showing that higher nurse turnover is associated with increased patient falls and other adverse outcomes.^26,39,40,41^ Specifically, RN turnover has been associated with higher rates of unassisted falls in medical-surgical units,^26^ underscoring the importance of stable and experienced nursing teams. These findings highlight the need to consider organizational characteristics and the work environment as factors associated with nurse turnover and important quality measures such as patient falls. Turnover may serve as a proxy for nurse characteristics that influence patient outcomes, such as experience and unit tenure.^42,43^ Additionally, prior research has suggested that effective onboarding and orientation may help with transition to practice and retention of newly licensed nurses.^44,45,46^

The SEM was used to explore whether nurse satisfaction may indirectly influence patient falls through turnover. Of the 7 job satisfaction items, nurse intent to stay showed a significant association with turnover. While all other practice environment elements contributed to the model, none were statistically significant. Our analysis builds on prior research that investigated different pathways between nurse practice environment and nursing emotional outcomes^47^ by showing both direct and indirect associations with patient outcomes. Nurse satisfaction as an indirect influence on patient falls through turnover is an important distinction to begin to address turnover. In this study, indirect referred specifically to the SEM pathway in which nurse job satisfaction and practice environment influenced patient falls indirectly through their association with turnover rather than as a direct association with patient falls. For example, the first stage of the SEM showed that the proportion of RNs who reported that they would not consider leaving their job was significantly associated with turnover, suggesting a pathway through which nurse practice environment engagement and retention could substantially influence patient safety outcomes. This association implies that higher levels of nurse intent to stay (ie, fewer nurses considering taking other jobs) may reduce turnover, which in turn could improve care, a critical factor in preventing adverse patient events such as falls.^26,40^ Reduction of nurse turnover is also key for hospitals as it not only may reduce adverse patient events but also may result in cost savings due to reduced costs of onboarding and training new nursing staff.^48^ Recent research has highlighted that lower nurse turnover rates were associated with fewer patient falls and may result in avoidable costs of $23 341 per unit for every 1000 patient-days.^27^ While research within the US context is limited, research outside the US has found that the nurse practice environment and workload is negatively associated with nurse-assessed quality of care.^49,50^ These results, paired with prior research, emphasize the need to further investigate key drivers of nurse turnover with additional nurse characteristics and perspectives, including the role of burnout.

This study also found that organizational and environmental characteristics are significantly associated with fall rates. Units categorized as surgical or medical-surgical had lower fall rates compared with medical units, suggesting differences in patient acuity, mobility, or care practices across unit types on fall outcomes.^51^ Hospitals with Magnet status and those with larger bed sizes are also associated with fewer falls, which is supported by prior research finding that Magnet-designated hospitals have experienced improvements not only in nursing workforce measures but also in patient outcomes, including patient falls.^21,36^ These findings suggest that supportive nurse practice environments may play a crucial role in preventing adverse events. Future research could explore organizational-level mechanisms, such as further exploring how subscales of the PES-NWI (eg, nurse resource adequacy, nursing foundations for quality care) contribute to other adverse outcomes (eg, hospital-acquired pressure injuries, ventilator-associated events, fall severity). Understanding which aspects of the practice environment are most protective may help guide targeted interventions at the unit and hospital levels.

These findings suggest that interventions to reduce inpatient falls should focus not only on patient-level risk factors but also on broader organizational context and workforce stability. Efforts to reduce nurse turnover, particularly in medical units, could have downstream effects on patient safety. Furthermore, the lower rates of patient falls and nurse turnover observed in Magnet hospitals point to the value of institutional commitments to nursing excellence and staff empowerment.

### Limitations

This study had several limitations. The observational design limited causal inference, and unmeasured confounding may have biased the results despite the use of SEM. The limited study period precluded longitudinal analysis. Turnover was measured as the percentage of RN and APRN staff who left their roles, preventing disaggregation by workforce type and limiting role-specific conclusions. Future research should examine RN and APRN turnover separately, as their rates and roles likely differ. Furthermore, while the dataset included hospitals nationwide, participation was incomplete, which may introduce selection bias. Multiple imputation and full information maximum likelihood assume that data are missing at random; thus, violations could have biased estimates. This analysis contained limited information on the demographics of patients and nurses, including nurse tenure, role, and patient acuity. Several key measures were available only at the unit level, constraining generalizability to individual patients or staff. Improved data on patient sex, including those who did not fall, and detailed acuity may have led to more nuanced results.

## Conclusions

This cross-sectional study found that nurse turnover may be an important and modifiable risk factor for patient falls. While most satisfaction and practice environment variables were not statistically significant, nurses’ intent to stay was associated with lower turnover, supporting the role of staff commitment in promoting patient safety. Future work should examine how interventions focused on the practice environment could improve nurse retention and support high-risk units in reducing falls and enhancing patient outcomes.

## Notes

Supplement 1. **eAppendix 1.** Hypothesized Association Among PES, Turnover, and Patient Outcomes **eAppendix 2.** Missing Data **eFigure 1.** SEM Conceptual Framework **eFigure 2.** Average Unit Turnover and Falls Per 1000 Patient-Days **eTable 1.** Regression and SEM Variables and Definitions **eAppendix 3. **Linear Regression Model Examining the Direct Relationship Between Aggregate PES-NWI Factors and Patient Falls **eTable 2.** Linear Regression Examining Increases in Patient Falls Per 1000 Patient-Days
eReferences.

Supplement 2.
Data Sharing Statement
