# Supplementary material for: Nurse Practice Environment, Job Satisfaction, and Turnover and Patient Falls
Source: JAMA Netw Open. 2025 Dec 29;8(12):e2551223. doi: 10.1001/jamanetworkopen.2025.51223 (PMC12750249; doi:10.1001/jamanetworkopen.2025.51223)
Supplement: Supplement 1. — eAppendix 1. Hypothesized Association Among PES, Turnover, and Patient Outcomes eAppendix 2. Missing Data eFigure 1. SEM Conceptual Framework eFigure 2. Average Unit Turnover and Falls Per 1000 Patient-Days eTable 1. Regression and SEM Variables and Definitions eAppendix 3. Linear Regression Model Examining the Direct Relationship Between Aggregate PES-NWI Factors and Patient Falls eTable 2. Linear Regression Examining Increases in Patient Falls Per 1000 Patient-Days eReferences. [file jamanetwopen-e2551223-s001.pdf]

Supplemental Online Content

Mauricio K, Bulger AL, Jurgens CY, Ruscitti B, Gregory KE, Bowser D. Nurse practice environment, job satisfaction, and turnover and patient falls. *JAMA Netw Open*. 2025;8(12):e2551223. doi:10.1001/jamanetworkopen.2025.51223

eAppendix 1. Hypothesized Association Among PES, Turnover, and Patient Outcomes

eAppendix 2. Missing Data

eFigure 1. SEM Conceptual Framework

eFigure 2. Average Unit Turnover and Falls Per 1000 Patient-Days

eTable 1. Regression and SEM Variables and Definitions

eAppendix 3. Linear Regression Model Examining the Direct Relationship Between Aggregate PES-NWI Factors and Patient Falls

eTable 2. Linear Regression Examining Increases in Patient Falls Per 1000 Patient-Days

eReferences

This supplemental material has been provided by the authors to give readers additional information about their work.

© 2025 Mauricio K et al. *JAMA Network Open*.

eAppendix 1. Hypothesized Relationship Among PES, Turnover, and Patient Outcomes

Nurse satisfaction and the quality of the nurse practice environment as measured by the responses to the NDNQI Rn survey may affect nurse turnover[1], job satisfaction[2], and patient outcomes.[3] Additionally, recent research shows that nurses faced increased workloads, longer hours, and heightened stress levels, leading to burnout and higher turnover rates.[2] Staffing shortages exacerbated these issues, leaving fewer nurses to monitor patients, which increased the risk of falls.[3,4] High-stress environments and lack of adequate resources negatively impact job satisfaction, with many nurses feeling undervalued and unsupported.[5] These factors collectively reduced nurse engagement and attentiveness, leading to more adverse outcomes including patient falls.[6] This analysis hypothesizes that a relationship exists in which turnover negatively affects patient falls and turnover itself is related to nurse job satisfaction and nurse practice environment. Our SEM model aims to test this indirect relationship.

eAppendix 2. Missing Data

A preliminary analysis using Little's MCAR test revealed that the data was not missing completely at random. Given this result, the more tenable Missing at Random (MAR) assumption was adopted, making FIML an appropriate and efficient method for estimating the model parameters.[7] Although item-level missingness for some PES-NWI subscales was substantial (>70%), this reflects the survey design (not all units contributed nurse survey responses in each quarter) rather than data quality issues. Hospital-level outcomes and covariates were largely complete (<20% missing). In this case the FIML approach is appropriate and effective and has been used extensively to yield unbiased estimates using SEM.[7-9]

eFigure 1. SEM Conceptual Framework

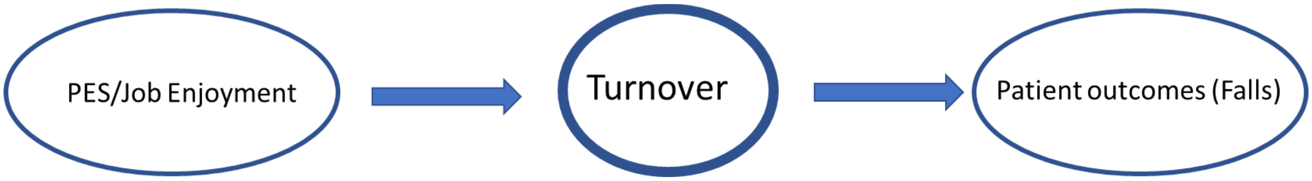

© 2025 Mauricio K et al. *JAMA Network Open*.

eFigure 2. Average Unit Turnover and Falls Per 1000 Patient Days

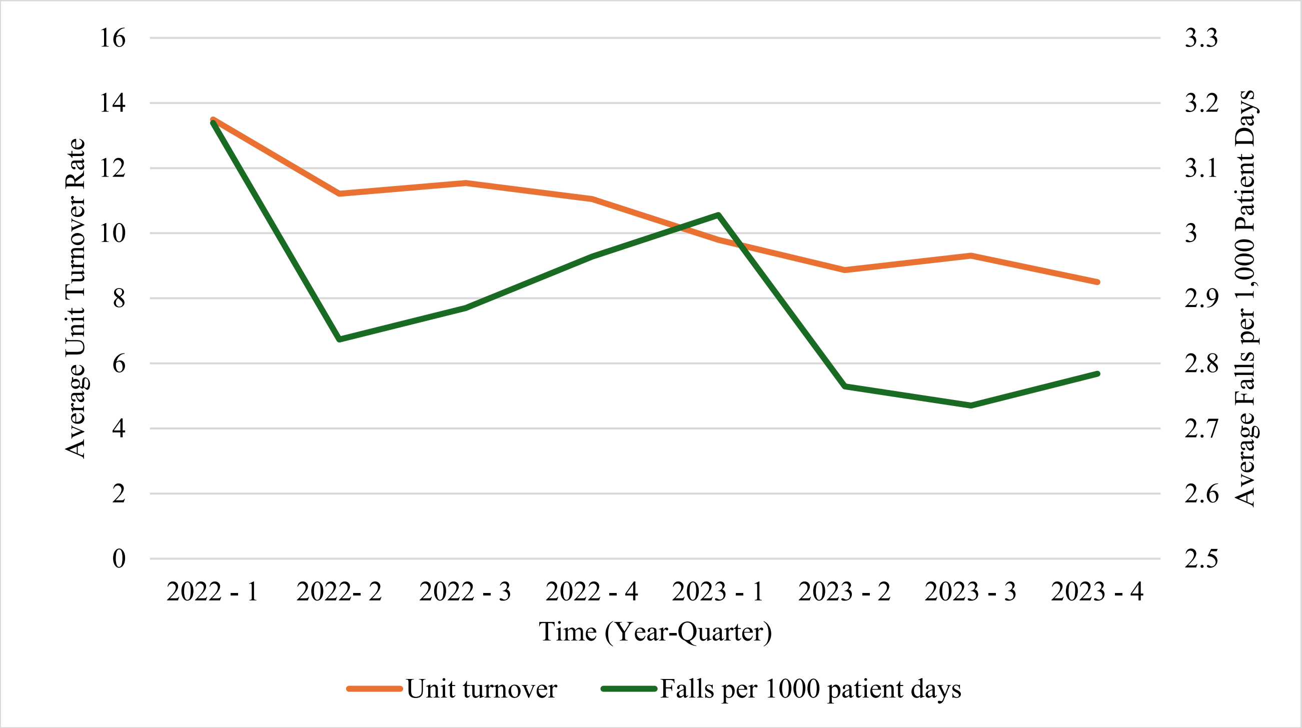

eTable 1. Regression and SEM Variables and Definitions

Variable	Definition
RN APRN Turnover	RN turnover within unit
Satisfaction	Nurse reported satisfaction as measured by RN survey
Average Age	Average age of patients who have fallen
F/M Ratio	Female to male ratio of patients who have fallen
Medical	Medical unit
Surgical	Surgical unit
Med/Surge	Medical and surgical combined unit
At risk share	Share of patients classified as at high fall risk
PES-NWI Resource availability	Aggregate measure of resource availability as reported by nurses
PES-NWI Quality Care	Aggregate measure of the foundations of nursing quality care as reported by nurses
Magnet Status	Magnet status of hospital
Bed category	Size of hospital as measured by number of beds

© 2025 Mauricio K et al. *JAMA Network Open*.

eAppendix 3. Linear Regression Model Examining the Direct Relationship Between Aggregate PES-NWI Factors and Patient Falls

eTable 2 below shows the results of a linear regression model examining the direct relationship between aggregate PES-NWI factors and patient falls. The results show that higher nurse turnover is significantly associated with higher fall rates ($p = 0.03$). A complete turnover of the staff (100% turnover) would be associated with 1 additional patient falls per 1,000 patient days. Alternatively, a unit with the national average nurse turnover of 18.4% would see an increase of 0.18 more falls per 1000 patient days compared to unit with zero turnover. Furthermore, regarding patient demographics, the sex ratio of patients who fall (female/male) has a strong positive correlation with fall rates ($p < 0.001$), showing that units having a higher proportion of female patients experienced more falls. In terms of unit and facility characteristics, unit type influences fall rates, with surgical and combined medical/surgical units experiencing fewer falls than medical units ($p < 0.05$). Hospitals with Magnet status also have fewer falls compared to those without ($p < 0.05$), and larger hospitals, measured by bed size, are associated with lower fall rates ($p < 0.001$). None of the included PES measurements, including nurse satisfaction, are significant in the linear regression models.

© 2025 Mauricio K et al. *JAMA Network Open*.

eTable 2. Linear Regression Examining Increases in Patient Falls Per 1000 Patient-Days

Variable	Coefficient	P-Value	95% Confidence Interval	
RN APRN Turnover	0.007	0.033	0.001	0.013
Nurse Job Satisfaction	0.042	0.814	-0.313	0.397
PES Sufficient Resources for Nurses	-0.06	0.760	-0.451	0.331
PES Foundations of Nurse Quality	-0.181	0.613	0.890	0.528
Average Age	0.001	0.739	-0.006	0.008
F/M Ratio	0.148	0.000	0.094	0.201
Medical				
Surgical	-0.516	0.000	-0.784	-0.247
Med/Surgical	-0.244	0.042	-0.480	-0.009
At-risk Share	1.336	0.000	1.119	1.554
Magnet Status				
Magnet - Applicant	0.152	0.399	-0.201	0.505
Magnet	-0.231	0.012	-0.410	-0.051
Hospital Size Category	-0.148	0.000	-0.219	-0.078
Constant	3.426	0.000	1.92	4.93

Note: Non-Magnet is the reference group for Magnet Status. As the Magnet application process may take a significant period of time, hospitals in the application process were considered separate from Magnet and Non-Magnet hospitals.

© 2025 Mauricio K et al. *JAMA Network Open*.

eReferences

1. Wei H, Horsley L, Cao Y, et al. The associations among nurse work engagement, job satisfaction, quality of care, and intent to leave: A national survey in the United States. *Int J Nurs Sci*. 2023;10(4):476-484.

2. Zink M, Pischke F, Wendsche J, Melzer M. Managing the work stress of inpatient nurses during the COVID-19 pandemic: a systematic review of organizational interventions. *BMC Nurs*. 2024;23(1):691. doi:10.1186/s12912-024-02358-1

3. NPSD Data Spotlight t. Patient Safety and COVID-19: A Qualitative Analysis of Concerns During the Public Health Emergency. *Agency Healthc Qual Patient Saf Rockv*. Published online 2021.

4. Aiken LH, Sloane DM, McHugh MD, Pogue CA, Lasater KB. A repeated cross-sectional study of nurses immediately before and during the COVID-19 pandemic: Implications for action. *Nurs Outlook*. 2023;71(1):101903. doi:10.1016/j.outlook.2022.11.007

5. Shan Y, Zhou X, Zhang Z, Chen W, Chen R. Enhancing the work engagement of frontline nurses during the COVID-19 pandemic: the mediating role of affective commitment and perceived organizational support. *BMC Nurs*. 2023;22(1):451. doi:10.1186/s12912-023-01623-z

6. Lavoie-Tremblay M, Gélinas C, Aubé T, et al. Influence of caring for COVID-19 patients on nurse's turnover, work satisfaction and quality of care. *J Nurs Manag*. 2022;30(1):33-43. doi:10.1111/jonm.13462

7. Enders CK, Bandalos DL. The relative performance of full information maximum likelihood estimation for missing data in structural equation models. *Struct Equ Model*. 2001;8(3):430-457. doi:10.1207/S15328007SEM0803_5

8. Graham JW. Adding Missing-Data-Relevant Variables to FIML-Based Structural Equation Models. *Struct Equ Model Multidiscip J*. 2003;10(1):80-100. doi:10.1207/S15328007SEM1001_4

9. Enders CK. *Applied Missing Data Analysis*. Guilford Publications; 2022.

© 2025 Mauricio K et al. *JAMA Network Open*.
