# Supplementary material for: Nurse Practice Environment, Job Satisfaction, and Turnover and Patient Falls
Source: JAMA Netw Open. 2025 Dec 29;8(12):e2551223. doi: 10.1001/jamanetworkopen.2025.51223 (PMC12750249; doi:10.1001/jamanetworkopen.2025.51223)
Supplement: Supplement 2. — Data Sharing Statement [file jamanetwopen-e2551223-s002.pdf]

Data Sharing Statement

Data

Data available: No

Additional Information

Explanation for why data not available: The data used in this study were obtained from the National Database of Nursing Quality Indicators® (NDNQI®), which is maintained by Press Ganey Associates LLC. Access to these data is restricted and governed by data use agreements. As such, the authors are not permitted to share the dataset publicly. Interested researchers may contact Press Ganey directly to inquire about data access.
